# *Plasmodium pitheci* malaria in Bornean orang-utans at a rehabilitation centre in West Kalimantan, Indonesia

**DOI:** 10.1186/s12936-022-04290-8

**Published:** 2022-10-03

**Authors:** Karmele Llano Sanchez, Alex D. Greenwood, Aileen Nielsen, R. Taufiq P. Nugraha, Wendi Prameswari, Andini Nurillah, Fitria Agustina, Gail Campbell-Smith, Anik Budhi Dharmayanthi, Rahadian Pratama, Indra Exploitasia, J. Kevin Baird

**Affiliations:** 1 IAR Indonesia Foundation – Yayasan Inisiasi Alam Rehabilitasi Indonesia (YIARI), Ketapang, West Kalimantan Indonesia; 2 International Animal Rescue, Uckfield, UK; 3 Department of Veterinary Medicine, Frei Universität, Berlin, Germany; 4 Present Address: Department of Wildlife Diseases, Leibniz Institute for Zoo and Wildlife Research, Berlin, Germany; 5 Center for Law and Economics, ETH Zurich, Zurich, Switzerland; 6 Research Center for Applied Zoology, National Research and Innovation Agency, Republic of Indonesia (BRIN), Cibinong, Indonesia; 7 Research Center for Biosystematics and Evolution, National Research and Innovation Agency, Republic of Indonesia (BRIN), Cibinong, Indonesia; 8 Department of Biochemistry, Faculty of Mathematics and Natural Sciences, IPB University, Bogor, Indonesia; 9 Biodiversity Conservation Directorate of the General Director of Natural Resources and Ecosystem Conservation, Ministry of Environment and Forestry of the Republic of Indonesia, Jakarta, Indonesia; 10 Clinical Research Unit-Indonesia, Faculty of Medicine, Oxford University, Universitas Indonesia, Jakarta, Indonesia; 11 Centre for Tropical Medicine and Global Health, Nuffield Department of Medicine, University of Oxford, Oxford, UK

**Keywords:** Malaria epidemiology, Great ape health, Orang-utan malaria, *Plasmodium pitheci*, Veterinary medicine, Clinical parasitology, Orang-utan conservation, Orang-utan disease

## Abstract

**Background:**

Plasmodial species naturally infecting orang-utans, *Plasmodium pitheci* and *Plasmodium silvaticum*, have been rarely described and reportedly cause relatively benign infections. Orang-utans at Rescue Rehabilitation Centres (RRC) across the orang-utan natural range suffer from malaria illness. However, the species involved and clinical pathology of this illness have not been described in a systematic manner. The objective of the present study was to identify the *Plasmodium* species infecting orang-utans under our care, define the frequency and character of malaria illness among the infected, and establish criteria for successful diagnosis and treatment.

**Methods:**

During the period 2017–2021, prospective active surveillance of malaria among 131 orang-utans resident in a forested RRC in West Kalimantan (Indonesia) was conducted. A total of 1783 blood samples were analysed by microscopy and 219 by nucleic acid based (PCR) diagnostic testing. Medical records of inpatient orang-utans at the centre from 2010 to 2016 were also retrospectively analysed for instances of symptomatic malaria.

**Results:**

Active surveillance revealed 89 of 131 orang-utans were positive for malaria at least once between 2017 and 2021 (period prevalence = 68%). During that period, 14 cases (affecting 13 orang-utans) developed clinical malaria (0.027 attacks/orang-utan-year). Three other cases were found to have occurred from 2010–2016. Sick individuals presented predominantly with fever, anaemia, thrombocytopenia, and leukopenia. All had parasitaemias in excess of 4000/μL and as high as 105,000/μL, with severity of illness correlating with parasitaemia. Illness and parasitaemia quickly resolved following administration of artemisinin-combined therapies. High levels of parasitaemia also sometimes occurred in asymptomatic cases, in which case, parasitaemia cleared spontaneously.

**Conclusions:**

This study demonstrated that *P. pitheci* very often infected orang-utans at this RRC. In about 14% of infected orang-utans, malaria illness occurred and ranged from moderate to severe in nature. The successful clinical management of acute pitheci malaria is described. Concerns are raised about this infection potentially posing a threat to this endangered species in the wild.

## Background

Human plasmodial parasites responsible for clinical malaria infect hundreds of millions of people annually, causing more than half a million deaths in areas of endemicity [1, 2]. Malaria is a major cause of morbidity and mortality in the tropics, affecting people of all ages, but especially in children and pregnant women [1–4]. With a wide geographic distribution of human plasmodial species throughout tropical and subtropical areas of the world [5–7], and over 100 countries where malaria is still considered endemic [7], malaria remains a priority focus for international efforts aimed at mitigating the harm done [8, 9].

Many dozens of species in the genus *Plasmodium* are known to naturally infect some mammals, birds, and reptiles [10–13]. Just four of those species are considered adapted to humans as primary intermediate hosts [14, 15], a relationship hereby referred to as natural, and all are considered pathogenic. A fifth malaria species also successfully infects humans but not as its natural host; *Plasmodium knowlesi*, a non-human primate (NHP) *Plasmodium* that occurs in macaques, but also infects humans [16, 17]. *Plasmodium knowlesi* malaria in humans is sometimes lethal, and in Malaysia today it is the overwhelmingly dominant cause of human malaria [11, 17–21].

The health effects of malaria in humans are well-known and have serious implications for human health among malaria-endemic nations [4, 5, 7, 11, 22]. NHP plasmodial species are highly diverse [11, 21, 23]. In the absence of studies focussing on malaria pathology in great apes [24, 25], the health effects of these NHP *Plasmodium* in their natural host is controversial, and questions remain regarding their potential pathogenicity [24, 26–33]. Natural infection with *Plasmodium*, sometimes with high prevalence, are widely reported in wild as well as captive populations of great apes within their range and are commonly considered harmless in their natural host with allegedly negligible clinical implications for infected individuals [12, 24, 26, 27, 29, 33–38]. However, cases of malaria illness have been reported in chimpanzees [30, 39] and in a gorilla (as reported by Dian Fossey in her book “Gorillas in the Mist”) [40]. Similarly for orang-utans, while malaria has been widely referred to as a benign infection or causing “little discomfort” [23, 29, 33, 34], there are some reports of serious illness. Dodd in 1913 reported a case of suspected malaria disease and mortality in an orang-utan infected with *P. pitheci* at a zoo in Sydney [23, 33]. More recently, other authors [28, 29, 41] have described a debilitating disease in orang-utans that courses with symptoms consistent with malaria infection and responds to anti-malarial drugs. At present, experienced veterinarians caring for wild rescued orang-utans at RRCs across their range, consider malaria a potentially serious illness capable of causing death (Fransiska Sulistyo, pers. comm.).

The novel discovery of a malaria parasite in NHP occurred over 100 years ago when Halberstaedter and Prowazek at a Berlin zoo housing an orang-utan originating from Borneo first described *P. pitheci* [11, 23, 33]. Subsequently, during the first half of the twentieth century, many other malaria species were discovered in African great apes [24, 40], and in 1972, another orang-utan malaria species, *P. silvaticum*, was described [33]. In the current century, the development of molecular techniques has allowed for the genetic characterization of *Plasmodium* pathogens from non-invasive samples from African great apes [11, 24, 32, 36–38, 40, 42, 43]. As such, malaria research on great apes has mainly focussed on parasite diversity, phylogeny and evolutionary history of African *Plasmodium* species [24, 40]. Meanwhile, molecular characterization of the Asian ape plasmodial species has lagged behind [11, 44, 45]. Both *P. silvaticum* and *P. pitheci* which were morphologically described as species long before the advent of molecular techniques, have yet to be genetically characterized. Consequently, there are no validated molecular probes for PCR-based identification of these two species.

A previous study on orang-utans at an RRC in Borneo [28, 29] attributed malaria infection to non-orang-utan plasmodial species: a human (*Plasmodium vivax*) and a macaque (*Plasmodium cynomolgi*) *Plasmodium* species [29]. This study also reported a higher prevalence of malaria infection in orang-utans rescued as pets over those living at the RRC [29] and hypothesized orang-utans as potential reservoirs for human malaria [29, 46]. This was the first report of potential human-orang-utan malaria parasite transmission. This is a plausible scenario since anthroponotic transmission of human malaria parasites has been widely reported for great apes and other NHPs [10–12, 32, 37, 40, 47]. Nevertheless, Reid’s findings were subsequently examined by Singh and Divis [46], who demonstrated that the *Plasmodium* species involved were neither *P. vivax* nor *P. cynomolgi.* As no morphological characterization was made in that study, the species involved remains unknown [44, 46], but *P. pitheci* or *P. silvaticum* are considered candidate pathogens in this case [48]. Nevertheless, despite the development of no further evidence, this “malaria-like” illness reported at RRCs has continued to be attributed to anthroponotic transmission [41].

Rescued wild orang-utans displaced from their forest habitat due to habitat fragmentation or the demand for illicit wildlife pet trade, are cared for at RRCs. The number of orang-utans at these RRCs represents an important proportion of the remaining wild population, and therefore, this illness could have serious implications for the conservation of the species. A large body of laboratory and clinical evidence was explored to examine possible causal relationships between *Plasmodium* spp. infection and potentially threatening illness in orang-utans. In doing so, in an attempt to optimize and validate the clinical management of infected orang-utans thresholds of parasitaemia, sickness and regimens of clinical management and chemotherapy leading to safe and full recovery were quantified.

## Methods

### Study site and subjects

The *Inisiasi Alam Rehabilitasi Indonesia* Foundation (IAR Indonesia) works under the Directorate of Biodiversity Conservation of the Ministry of Environment and Forestry (MoEF) of the Republic of Indonesia to operate a rescue and rehabilitation centre (RRC) for Bornean orang-utans in Ketapang, West Kalimantan, Indonesia, the IAR RRC. This centre began operations in 2009 and has since rescued over 250 orang-utans. The observational study reported herein was reviewed and approved by the Ethics in Research Commission of the National Research and Innovation Agency of the Republic of Indonesia (Experimental Use of Animals, Letter Number 86/CI/2021; 21 November 2021).

The data used in this study was collected retrospectively (2010–2016) and prospectively (2017–2021). The retrospective study consisted entirely of a review of medical records for instances of symptomatic confirmed malaria illness. During the prospective study, a total of 131 orang-utans (60 females and 71 males) were observed. Among those, 49 were lost to observation—48 by being reintroduced into the wild, and one orang-utan that died—and 29 newly arrived at IAR RRC.

#### Routine management and care procedures

All orang-utans arriving at IAR RRC spend a minimum of 60 days in quarantine. During this time, all undergo medical checks as part of their medical quarantine procedure. Upon completion of the quarantine period, healthy orang-utans are transferred to the rehabilitation areas or socialization cages, where they join with conspecifics of approximately the same age and size. Some orang-utans are deemed unsuitable for rehabilitation and release, and they become permanent residents. These include: older juveniles or adult orang-utans that matured in captivity (with private owners); those who spent longer than eight to nine years in that condition; or those with disabilities or diseases that make survival in the wild unlikely. All other orang-utans undergo a ‘rehabilitation’ process that ends with reintroduction into suitable wild protected forested areas.

The rehabilitation activities take place in semi-natural secondary forested areas, delimited by artificial canals and electric fences. All the orang-utans temporarily housed on these islands are grouped by size and/or age. Each area, depending on its size (approximately 10–20 ha), can accommodate between 10 and 20 orang-utans where they have contact with wild animals such as macaques, leaf monkeys, proboscis monkeys, and a variety of other mammals, birds and reptiles, as well as non-vertebrate fauna. After rehabilitation, orang-utans are reintroduced into a protected natural habitat designated for this purpose. Intense post-reintroduction monitoring continues for as long as possible to evaluate their natural behaviours and their capacity to adapt to the environment.

The health of all orang-utans managed by IAR RRC is closely monitored daily by a team of veterinarians, veterinary assistants and animal keepers. Biological samples are collected only for medical purposes from healthy animals during routine medical check-ups or when orang-utans show any signs of illness. Medical records are kept for each orang-utan and every medical procedure is recorded both in a hard copy format (paper files) and soft copy format (using FileMaker® database).

#### Sample collection and classification

As part of routine medical checks conducted by the staff veterinarians, a total of 1783 blood samples from a cohort of 131 orang-utans housed at IAR RRC were collected and analysed at the in-house laboratory facility between January 2017 and December 2021. Blood samples were collected from orang-utans during either manual restraint or incident to clinically indicated anaesthesia. Samples were classified per sampling purpose of each screening event:

(i) *Routine medical health checks*: (a) annual health checks; (b) quarantine procedures on arrival and prior release; or (c) health check-ups conducted to monitor the health of the population (cross-sectional sampling);
(ii) *Health monitoring for diagnostic purposes of inpatients* during any illness (except malaria) and during recumbence periods**;**
(iii) *Any medical or handling procedure requiring anaesthesia*
(iv) *Monitoring the health of patients presenting Plasmodium spp. patent infections:* routine consecutive screening in those individuals known to present patent malaria infection either symptomatically or asymptomatically, for the monitoring of haematology values and/or parasitaemia levels.

### Sample analysis

#### *Plasmodium* spp. detection

##### Microscopy

Fresh blood samples were collected by venipuncture and immediately prepared in thick and thin blood smears on glass slides. Smears were dried, thin smears were dipped in absolute methanol, and both thin and thick smears then stained for 15–20 min using Giemsa’s solution at 10%. The slides were then examined by trained personnel using a light microscope (Olympus® CX22LED) set at 1000× oil immersion optics and analysed for the detection of *Plasmodium* spp. by microscopic examination. If one or more sexual or asexual forms of *Plasmodium* spp. parasite was detected, the sample was classified as positive.

##### Molecular detection by conventional PCR and Real Time-PCR

DNA was extracted directly from fresh blood in EDTA, or from frozen whole blood in EDTA stored at − 80 °C using a commercially available kit: PureLink® Genomic DNA Mini Kit (Invitrogen, ThermoFisher®, USA), following the protocol provided with the kit. Extracted DNA was used directly for PCR analysis, or stored at − 80 °C.

##### Real time-PCR

Blood samples were tested by q-PCR on a Genesig q16® Real Time-PCR machine (Primerdesign® Ltd, UK) using a commercially available kit: *Plasmodium* (all species) Genesig® Easy kit (Primerdesign® Ltd, UK) following the standard protocol provided with the kit. This kit targets the 18S ribosomal gene achieving a broad-based detection profile for several species of *Plasmodium*.

##### Conventional PCR

Fresh and stored blood samples (frozen at − 80 °C) were analysed on a conventional gel-based nested PCR. For the detection of *Plasmodium* spp. published primers targeting highly conserved regions of the 18S small subunit ribosomal RNA genes were used (Table 3) following this protocol [29] (Table 1).

**Table 1:** Nested PCR protocol

Step	Nested 1 PCR		Nested 2 PCR	
	Primers: PLU1 & PLU5		Primers: PLU3 & PLU Cal 2	
	Product size: ~ 1640 bp		Product size: ~ 1500 bp	
	Temperature (°C)	Time	Temperature (°C)	Time
Initialization	95	5 min	95	5 min
Replication (40 cycles)				
Denaturation	96	5 s	96	5 s
Annealing	55	5 s	55	5 s
Extension	68	50 s	68	45 s
Final extension	72	1 min	72	1 min
Final hold	4	∞	4	∞

### Plasmodium species identification

#### Microscopy

Species identification was conducted by morphologic characterization of parasites infecting intact red blood cells on thin blood films using light microscopy [15, 21]. Identification was performed by applying parasite species descriptions of those known to naturally occur in primates from Coatney et al. [23] and those occurring specifically in Bornean orang-utans [33].

#### Molecular identification by conventional PCR

For identification of the species a set of positive samples were analysed by gel-based nested PCR using eight species-specific inner primers for different human and NHP species of *Plasmodium* (Table 3) following the protocol in Table 2.

**Table 2:** Nested 1 PCR protocol

Nested 1 PCR	Primers: PLU3 & PLU4	
Step	Temperature (°C)	Time
Initialization	94	5 min
	55	30 s
	72	2 min
Replication (29 cycles)		
Denaturation	94	30 s
Annealing	55	30 s
Extension	72	1 min 30 s
Final extension	72 °C	5 min

The protocol of the nested 2 PCR was the same as nested 1 PCR except for the number of cycles (35 cycles) and the annealing temperatures for species specific primers which was as follows: for *Plasmodium falciparum, Plasmodium ovale, Plasmodium malariae* was 58 °C; for *P. vivax* was 65 °C; for *Plasmodium inui, P. cynomolgi* was 60 °C; and for *P. knowlesi* 62 °C (Table 3).

**Table 3:** Primers for gel-based PCR [17, 49, 50]

*Plasmodium* species	Primer code	Sequence
*Plasmodium* sp. Genus Specific	PLU1	TCA AAG ATT AAG CCA TGC AAG TGA
	PLU3	TTT TTA TAA GGA TAA CTA CGG AAA AGC TGT
	PLU4	TAC CCG TCA TAG CCA TGT TAG GCC AAT ACC
	PLU5	CTT GTT GTT GCC TTA AAC TTC
	PLU6	TTA AAA TTG TTG CAG TTA AAACG
	PLU Cal 2	ACA CAW RGT KCC TCT AAG AAG C
*P. falciparum* (~ 206 bp)	rFAL1	TTA AAC TGG TTT GGG AAA ACC AAA TAT ATT
	rFAL2	ACA CAA TGA ACT CAA TCA TGA CTA CCC GTC
*P. vivax* (~ 205 bp)	rVIV6	TAA CGC CGT TAG CTA GAT CCA CAA GG
	rVIV7	CTG TAG TAT TCA AAA CGC GCA ATG CTG
*P. malariae* (~ 145 bp)	rMAL1	ATA ACA TAG TTG TAC GTT AAG AAT AAC CGC
	rMAL2	AAA ATT CCC ATG CAT AAA AAA TTA TAC AAA
*P. ovale* (~ 419 bp)	rOVA1	ATC TCT TTT GCT ATT TTT TAG TAT TGG AGA
	rOVA4	ACT GAA GGA AGC AAT CTA AGA AAT TT
*P. knowlesi* (~ 279 bp)	Kn1f	CTC AAC ACG GGA AAA CTC ACT AGT TTA
	Kn3r	GTA TTA TTA GGT ACA AGG TAG CAG TAT GC
*P. inui* (~ 479 bp)	PinF5	GTA TCG ACT TTG TGC GCA TTT TTC TAC
	INAR3	GCA ATC TAA GAG TTT TAA CTC CTC
*P. cynomolgi* (~ 137 bp)	CY2F	GAT TTG CTA AAT TGC GGT CG
	CY4F	CGG TAT GAT AAG CCA GGG AAG T
*P. coatneyi* (~ 505 bp)	PctF1	CGC TTT TAG CTT AAA TCC ACA TAA CAG AC
	PctR1	GAG TCC TAA CCC CGA AGG GAA AGG

### Parasite load in blood

The number of sexual and asexual parasites in red blood cells (RBC) was counted by examining Giemsa-stained thick smears by 1000× light microscopy using oil immersion until a total of 200 leukocytes (WBC) had been counted [51, 52]. This number was converted to parasites per microlitre (par/µL) of blood by multiplying it by the actual leukocyte levels when contemporaneous haematology data was available, and divided by 200 [21, 53], or multiplied by 60 if WBC data was not available (assuming an average WBC count in orang-utans of 12,000/μl—see Table 4).

**Table 4:** Blood parameters range values for healthy orang-utans at IAR RRC

Analyte	Units	Range of normal values in orang-utans at IAR	Mean	SD	Median
Red blood cell count (RBC) (automated)	10^12^/L	3.11–6.29	5.0	0.72	5.1
Haemoglobin (HGB) (automated)	g/dL	8.2–13.4	10.7	1.31	10.7
Haematocrit (HTO) (automated)	%	23.8–43	34.1	4.34	34.2
Platelet count (PLT) (automated)	10^9^/L	70–385	225.4	72.5	215.5
White blood cell count (WBC) (automated)	10^9^/L	4.7–18.4	11.3	3.73	11.8

### Blood haematology analysis

Analysis of haematology values including red blood cell count (RBC), leukocytes count (WBC), haemoglobin (HGB), haematocrit (HCT) and platelets (PLT) was conducted by collecting fresh blood samples by venepuncture and transferring them to EDTA coated tubes. These blood samples were directly analysed using an in-house Abaxis VetScan® HM5 automated haematology analyser. Haematology ranges were obtained by analysing 758 samples corresponding to 150 healthy orang-utans at the IAR RRC. The sampled population comprised rehabilitant, wild and pet orang-utans in the range of ages from infant, juvenile, sub-adult and adult [54] (Table 4). For this study and based on the range values obtained here, anaemia was defined as HGB below 8.2 mg/dL, leukopenia as WBC below 4.7*10^9^/L, and thrombocytopenia as PLT lower than 70*10^9^/L of blood.

### Epidemiological analysis

The prospective epidemiological analysis of orang-utans at the IAR RRC commenced in 2017. The initiation of surveillance and follow-up in each individual occurred opportunistically, i.e., when permitted by accessibility of a blood sample for analysis and at random routine tests conducted in the population. Among the 131 orang-utans involved, the number of malaria independent examinations (those examinations that were conducted not dependent of malaria infection status) ranged from 1 to 23 with a median of 8. As there were newly arrived rescued orang-utans added and others that were removed from the study population during the surveillance period, the initiation and finalization of testing varied widely for each individual. The results of these examinations were classified as negative, positive asymptomatic, or positive symptomatic.

Incidence rate (IR) per 100 orang-utan—year was obtained reviewing data from inpatient medical records between 2010 and 2021 and was defined as the number of new cases of clinical malaria in a year divided by the total orang-utan-time at risk. IR was calculated using an approximate denominator based on the total number of disease-free animals at the start of the time period, from which ½ of the withdrawn animals were subtracted and ½ of the new additions were added [55].

### Malaria definitions

The following definitions were developed based on published literature for human malaria [51, 52, 56] and previous experience treating malaria cases in orang-utans at IAR RRC:

- *Asymptomatic malaria* defined as the presence of *Plasmodium* spp. parasites in blood (patent infection) in the absence of fever [15, 53, 56–59] and any other observable signs of disease—we also included here cases of “asymptomatic anaemia” (anaemia in a healthy individual, in the absence of fever or other observable signs)
- *Clinical malaria* (symptomatic) cases were categorized as follows:

“Acute uncomplicated malaria” defined as symptomatic parasitaemia in the presence of fever and other symptoms and in the absence of signs of severity and/or evidence of vital organ dysfunction or the presence of any sequela post-infection [2];

“Chronic uncomplicated malaria” in cases when parasitaemia and anaemia persist for prolonged periods;

“Mild malaria” as clinical cases of uncomplicated self-limiting illness (which always included fever) with spontaneous decrease in parasitaemia and with mild symptoms that resolve without antimalarial treatment.

- *Severe malaria* was defined as malaria infection in which the patient presents vital organ impairment [51, 53], including severe neurological signs (coma or mental status impairment, prostration, multiple convulsions and other neurological impairments).

### Clinical case review

A retrospective review of all medical histories of febrile illnesses recorded as acute malaria between January 2010 and December 2021 was conducted. Medical history files of orang-utan patients at IAR RRC included data on anamnesis, results of all diagnostic procedures conducted, daily evolution notes, treatment administered, prognosis, differential and final diagnosis (when known). During the review, case reports in which microscopy-demonstrated malaria was confirmed as the diagnosis were selected for further investigation of the course of disease and treatment. The criteria for case selection were as follows: (1) axillary temperature > 38 °C with other symptoms recorded; (2) haematology values recorded; (3) malaria confirmed with numbers of parasites in blood recorded; and (4) responses to anti-malarial therapy recorded.

Evaluation of the response to treatment was attempted using information on the evolution of the severity of symptoms, recording the length of the course of the illness, and by comparison of pre- and post-treatment clinical variables (haematology and parasitaemia) for all the medical records for which this data was available. Any possible correlation or association with level of parasitaemia and signs of illness was examined, i.e., fever, anaemia, thrombocytopenia, and leukopenia. The presence of any sequela was evaluated and when possible, any known recurrence of illness was recorded (recurrence of asexual parasitaemia after treatment due to relapse, recrudescence or a new infection [57]).

### Effect of anti-malarial treatment in clinical cases of malaria

#### Anti-malaria drugs and posology

The anti-malarial treatment selected to treat malaria in orang-utans at IAR RRC were based on guidance for therapies used in human malaria [14, 57]:

- Oral administration of quinine sulphate at 10 mg of salt/kg of body weight 3 times a day (Q8H) in combination with doxycycline 2.2 mg/kg in a single dose per day (Q24H) for 7 days;

And artemisinin-based combination therapy (ACT):

- Oral administration (PO) of artesunate (4 mg/kg BW) combined with amodiaquine (10 mg/kg BW) once a day (Q24H) for 3 days
- Intramuscular administration of artemether at 3.2 mg/kg on an initial dose on the first day, followed by 1.6 mg/kg single dose a day (Q24H) for 6 days combined with doxycycline at 5 mg/kg (Q24H) for 7 days.

In human malaria, a correlation between parasitaemia loads and morbidity and severity of clinical malaria illness has been demonstrated [4, 15, 60]. To determine whether the number of parasites correlated with clinical signs of malaria in orang-utans, we statistically analysed parasite load and blood values of HGB (Fig. 5), PLT and WBC.

#### Statistical analysis

Statistical analysis was conducted with the R statistical package (R Core Team 2020). For comparison of pre and post treatment clinical values, the within-subject post-treatment value was subtracted from the pre-treatment value, and this was tested for a non-zero mean with a one-sample t-test. For reported correlations (between parasitaemia and haematology values, and between parasitaemia and haematology values pre and post treatment), the statistical significance was computed as the p-value for a least-squares linear regression (that is, using a t-distribution with n-2 degrees of freedom). Due to the small sample sizes, p values were not adjusted for multiple comparisons.

## Results

### Diagnosis

#### PCR findings

The sensitivity and specificity of the microscopy testing was tested obtaining a 100% specificity of the microscopy test when compared to nested or Real-Time PCR. Sensitivity of the malaria microscopy test was 78.17% (95% CI 72.40–83.94%; n = 197) compared to Real-Time PCR and 83.96% (95% CI 80.81–93.11%; n = 115) compared to nested PCR. Microscopy diagnostics were compared to conventional PCR and Real-Time PCR for the detection of *Plasmodium* spp. in this cohort; 13.04% of microscopy-negative samples were found to be positive by conventional nested PCR (95% CI 6.89–19.20%; n = 115) and 21.83% positive by Real-Time PCR (95% CI 16.06–27.60%; n = 197). Approximately 20% of PCR-confirmed infections were not detected by light microscopy. In the analyses that follow, only microscopic diagnostics were considered because these were complete across orang-utan examinations over the years of observation. Routine PCR diagnosis of malaria is not practical at this RRC.

A subset of 3 microscopy-positive and PCR-positive samples were examined using nucleic acid molecular testing. PCR probes specific for *P. falciparum, P. vivax, P. malariae, P. ovale, P. knowlesi, P. cynomolgi,* and *P. inui* were applied (Table 3). All tested positive for *Plasmodium* spp. but did not yield positive findings for any of these 8 species.

#### Morphology

**Fig. 1:**
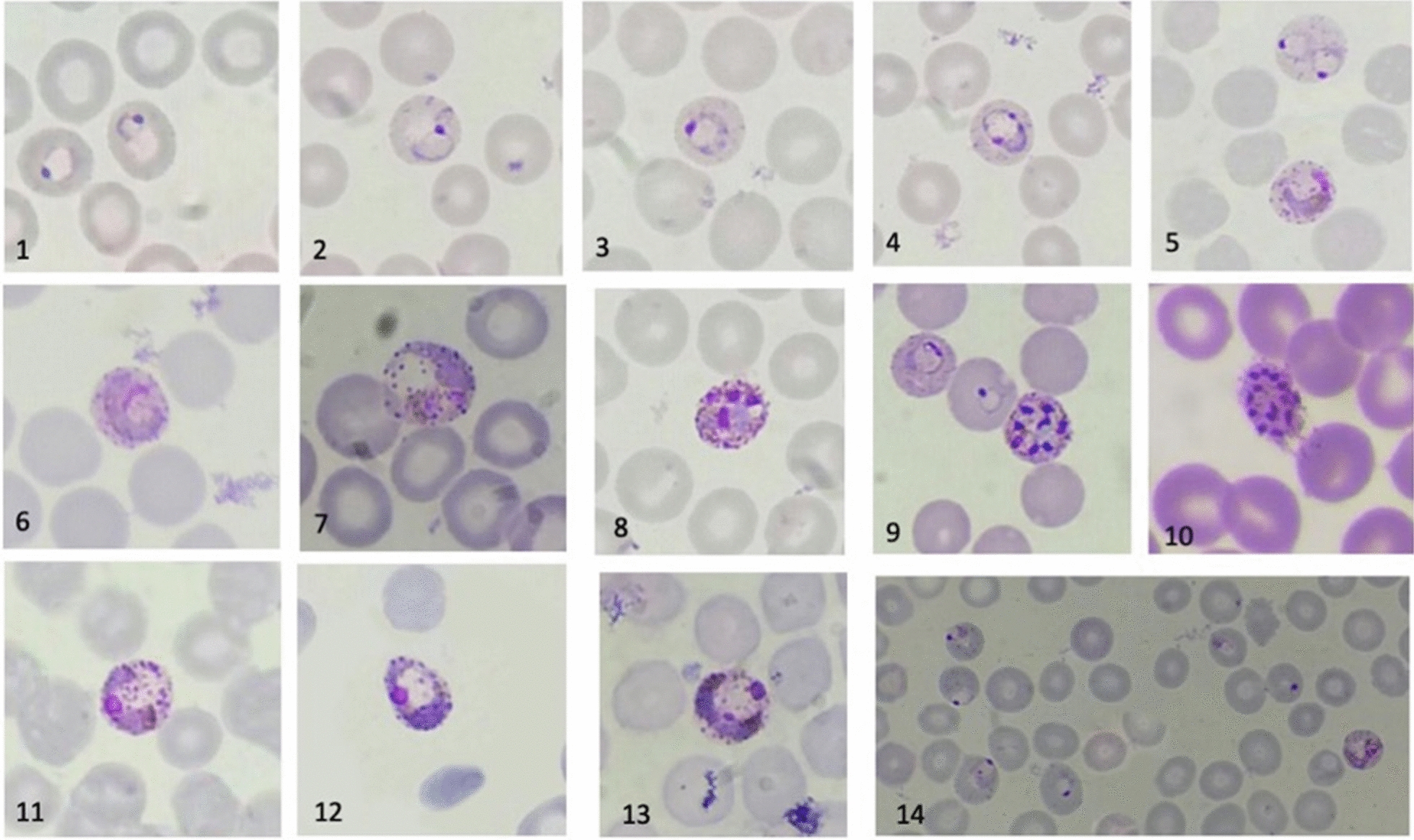
Composite of development of *Plasmodium pitheci* consistently observed among infected orang-utans at IAR RRC during the study period: Early ring stages (1–3), maturing trophozoites (4, 5), mature trophozoites (6, 7), maturing schizonts (8, 9), mature schizont (10), and microgametocytes (11–13). Note the absence of enlarged red blood cells at any stage of development and dispersed, un-clumped hemozoin through latter development. These features, along with < 15 merozoites within mature schizonts are the basis of the morphological diagnosis of *P. pitheci*. Panel 14 illustrates a thin smear from the presentation of Case 9 (Bunga) with a parasitaemia of over 20,000/µL blood and an acute febrile illness

A diagnosis of *P. pitheci* as the infecting species was based on the morphological features of the forms infecting red blood cells being wholly consistent with the descriptions of this species by Coatney et al. [23] and Peters et al. [33] (Fig. 1). Specifically, infected red blood cells were not enlarged at any stage of development, unlike those of *P. sylvaticum, P. cynomolgi, P. coatneyi, Plasmodium simiovale, Plasmodium fieldi, Plasmodium eylesi, P. vivax,* or *P. ovale*. Among the malaria parasites of primates that do not enlarge infected red blood cells, those that have mature schizonts without clumped pigment include *P. pitheci*, *Plasmodium youngi*, and *Plasmodium hylobati*. Among those three species, the mature schizonts of *P. youngi* and *P. hylobati* sometime exceed 14 merozoites, whereas those of *P. pitheci* do not. Mature schizonts with more than 14 merozoites were never observed. In summary, the observed plasmodial species infecting the orang-utans never enlarged red blood cells, did not form clumped pigment, and did not exceed 14 merozoites within mature schizonts. The morphology being consistent with only one of the two plasmodial species known to naturally infect Bornean orang-utans, led to the diagnosis of *P. pitheci* (Fig. 1). No other morphological or PCR evidence of any other species infecting the orang-utans was detected at IAR RRC during the period 2017–2021. Moreover, no case of acute malaria occurred among the staff of IAR RRC during this time.

### Epidemiology 

#### Retrospective study of acute malaria

**Fig. 2:**
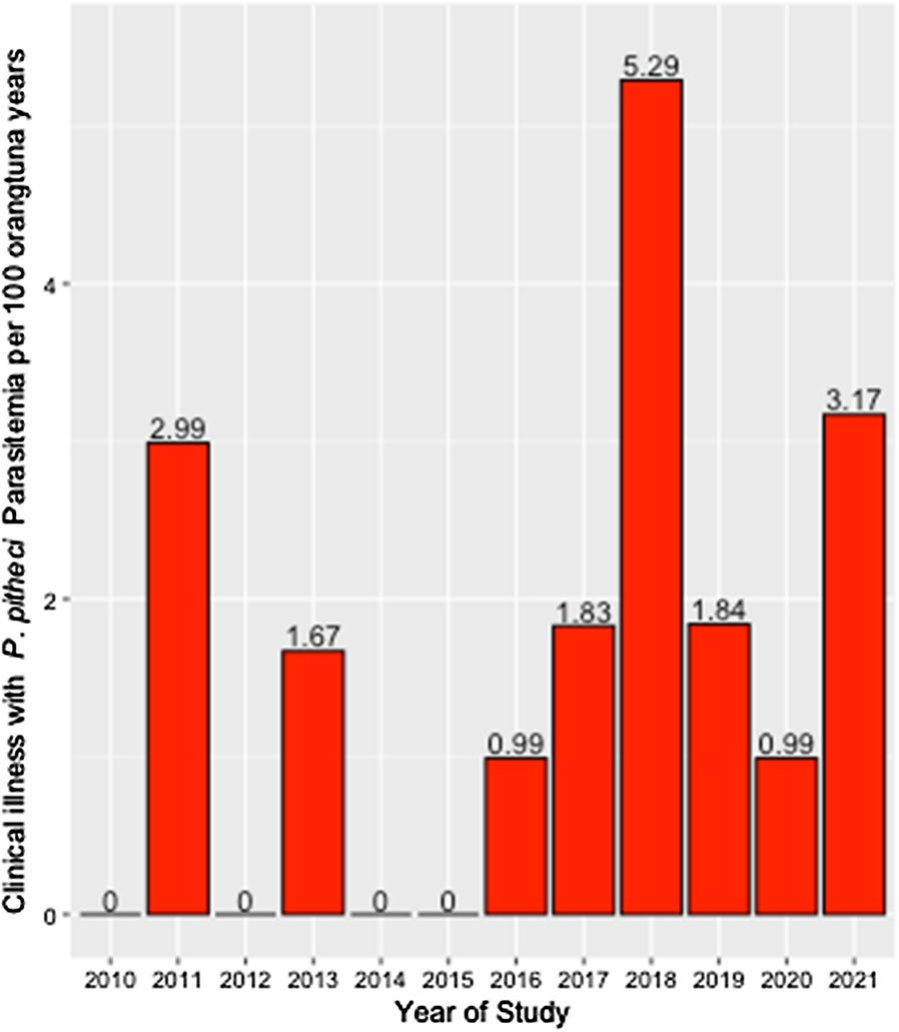
Annual incidence of clinical illness with *P. pitheci* parasitaemia among those residing at IAR-RRC, West Kalimantan, Indonesia

Inpatient medical records data were used to identify all clinical malaria cases detected since the beginning of the operations of the centre, between January 2010 and December 2021 following the criteria defined in the methods. A total of 17 cases of clinical malaria (symptomatic) cases were recorded comprising an overall incidence rate (IR) of 3.25 cases per 100 orang-utan-years for the entire period. Figure 2 illustrates the annual IR over the study period.

#### Prospective active case detection

**Fig. 3:**
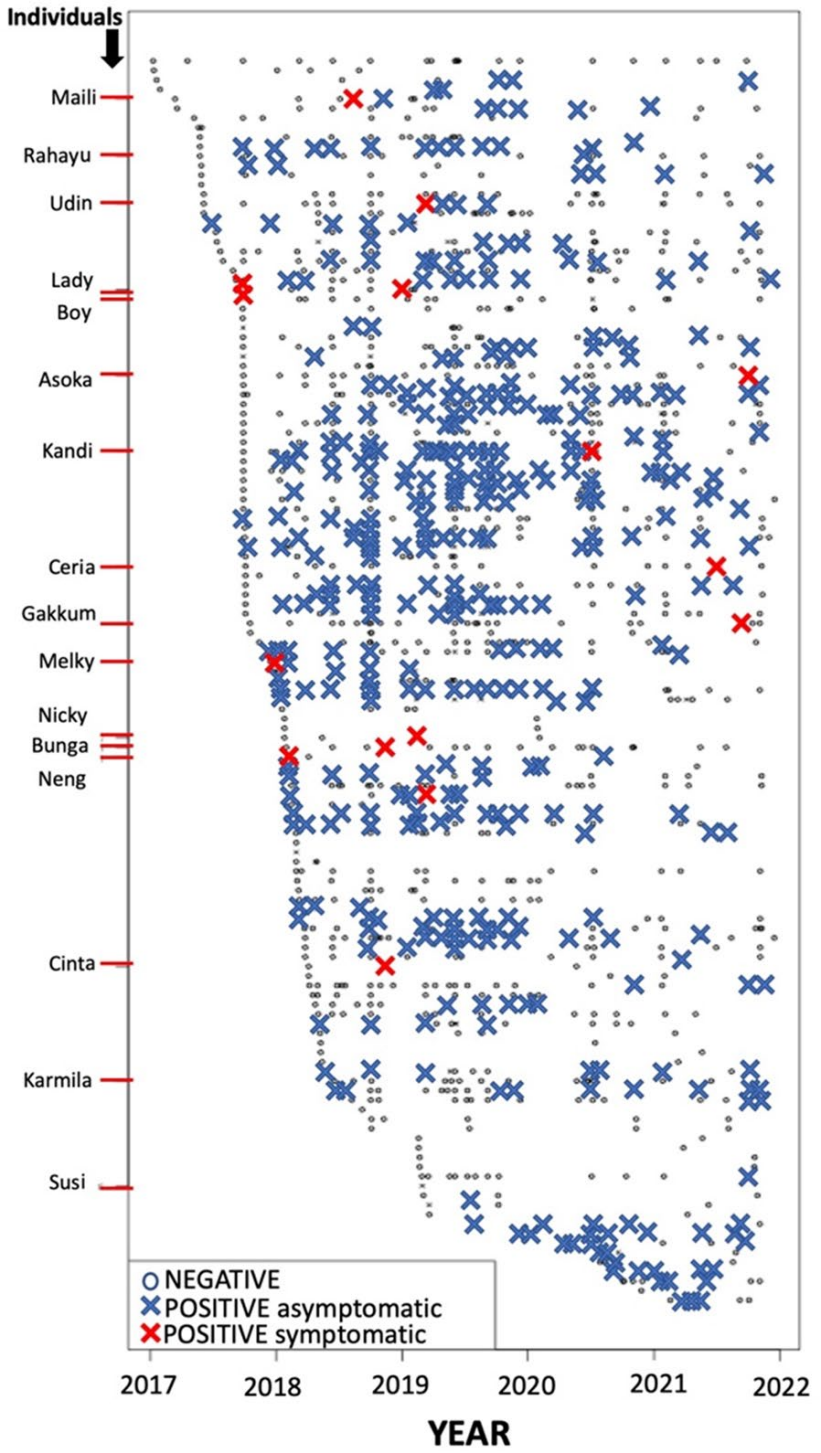
Prospective microscopic malaria surveillance findings among 131 orang-utans (each row is an individual) from January 2017 to December 2021. The named individuals were those progressing to a state of illness. The events of illness in Rahayu, Karmila, and Susi occurred prior to 2017

A total of 1783 samples were collected and microscopically examined for *Plasmodium* spp. infection during the surveillance period between January 2017 and December 2021 including those samples collected from orang-utans suffering clinical malaria. For the epidemiology analysis, only the first sample tested of each screening event was considered (defined by the screening purpose) for each individual. A total of 1255 blood samples from 131 orang-utans that were assessed for malaria during this period, and among them, 466 samples (37%) and 89 individuals (68%) were found positive for *Plasmodium*, all of which presented morphologic features consistent with *P. pitheci*. Figure 3 illustrates these findings.

### Clinical findings

#### Asymptomatic malaria

During 2017–2021, 76 of the 89 (85%) orang-utans found positive for malaria experienced no signs of illness despite weeks, months, and in some cases years of intermittent or sustained positivity (Fig. 3). 27 blood samples (6% of 444 positive samples) from 21 orang-utans exhibited parasitaemia > 3000/µL blood, and 17 of those samples (4%) from 15 orang-utans had a parasitaemia > 4000/µL. Amongst the individuals with blood parasitaemia > 3000/µL, 5 (19%) had mild to moderate anaemia, but showed no other sign of illness. In all of these cases, even though infections did not clear completely, parasite levels in blood decreased without the use of antimalarial therapies.

####  Clinical malaria

#####  Mild malaria:

Case 1 (Udin): A juvenile male orang-utan (approximately 5 years old) under rehabilitation was reported feverish and lethargic. He was brought to the clinic for further examination. His temperature was 39 °C. Microscopic examination of a blood sample revealed patent parasitaemia of *P. pitheci* > 32,000/µL. He also presented normocytic normochromic anaemia and thrombocytopenia, but no other specific finding was reported. He received antipyretics and remained in observation. The following hours he was reported bright, alert, and with good appetite, fever remitted within 48 h and parasite numbers in blood trended downwards without the need for any anti-malarial treatment. No further signs of illness were reported.

Case 2 (Kandi): A juvenile (4–5 years old) female orang-utan presented with fever (39.2 °C), normocytic normochromic anaemia and thrombocytopenia, but she remained active and with good appetite, and no other symptoms. Blood examination revealed *P. pitheci* parasites at a density of 16,600/µL. During observation fever remitted with the use of antipyretics alone, and the parasites in blood spontaneously decreased in number (although they did not clear completely), and her condition returned to normal within 48 h.

Case 3 (Boy): A juvenile male orang-utan of approximately 4 years of age while undergoing rehabilitation at the RRC, had presented a history of persistent weight loss, frequent illness and chronic microcytic hypo-chromic regenerative anaemia that did not respond to iron or vitamin supplements. He was presented to clinic suffering appetite loss, inactivity, and “looking pale” according to his keeper. Further examination revealed a fever (39 °C), diarrhoea and dehydration, and a blood profile of anaemia and leukopenia. Microscopic faecal examination revealed *Balantidium* spp. infection, therefore the patient was administered a course of metronidazole. He also received supplements to help resolve persistent anaemia. After the course of antibiotics, diarrhoea had resolved, however, his blood profile still revealed persisting anaemia and leukopenia, and a blood film examination revealed *P. pitheci* parasites > 12,000/µL. ACT was administered and improvement of the clinical and laboratory picture was nearly complete within five days. After this clinical episode, and during the whole period of surveillance (for over 4 years), no further anaemia or any other related medical problem was reported in this orang-utan.

Case 4 (Karmila): A juvenile (approximately 4–5 years) female orang-utan undergoing rehabilitation presented at clinic with a fever (39.6ºC), lethargy, anorexia and a patent *Plasmodium* spp. infection. She was administered IV fluids and other supportive therapy, and quinine/doxycycline combination. She fully recovered within seven days of treatment administration.

Case 5 (Gakkum): A juvenile male was brought to the clinic experiencing onset of fever (38.6 °C). He was active, had a good appetite and no other symptom was recorded. His blood sample was positive plasmodia at 7623/µL blood. He remained under observation. During the ensuing 5 days, he would peak a fever in the late afternoon/early evening hours (up to 39.9 °C) for which he received antipyretics. Although his general condition remained stable, his blood haematology values (HGB and PLT) started dropping. On day 6 after the onset of the fever, a normocytic normochromic anaemia was diagnosed while PLT values continue dropping and parasite numbers in blood climbed above 30,000/µL. His condition began deteriorating with conspicuous lethargy and inactivity. ACT was then administered and his condition began improving within 24 h, and after 4 days he had fully recovered.

Case 6 (Asoka): a lethargic juvenile male orang-utan presented to the clinic with onset fever (38.3 °C). He was otherwise bright, responsive and with good appetite. His blood showed normocytic normochromic anaemia and parasitaemia of *P. pitheci* at 17,500/µL. He was monitored for 3 days while his blood values (HGB, PLT and WBC) steadily dropped and his fever would spike in the afternoon. Nonetheless, he remained active and with good appetite. He was only administered antipyretics. On the 4th day after the onset of fever, his blood values remained dropping down, his temperature reached 39.8ºC and he presented more lethargic and unresponsive. He was administered ACT therapy. Within 24 h fever remitted and his condition started to improve, eventually resulting in an uneventful full recovery.

Case 7 (Lady): A sub-adult female orang-utan of approximately 9 years of age undergoing rehabilitation, presented with a high fever (40 °C), anaemia, thrombocytopenia and leukopenia. Microscopic examination of a blood sample revealed an infection of *P. pitheci* (> 26,000/µL). She was administered ACT and supportive therapy (including IV fluid therapy). Her fever remitted after 48 h and she had completely recovered by the end of the treatment.

Case 8 (Lady): 14 months after her first malaria episode in 2017, this sub-adult female orang-utan (10 years old) was again reported to be anorexic and lethargic. She presented to clinic with fever (39.7ºC), anaemia, thrombocytopenia and a *P. pitheci* parasitaemia at 11,080/µL of blood, although she remained active and bright. She was started on ACT and provided supportive therapy. Signs of recovery were evident within 24 h and no further sign of the illness was reported.

Case 9 (Bunga): A sub-adult female orang-utan around 11 years of age was being monitored in the wild after having been reintroduced 6 weeks earlier. She was found to be inactive, anorexic and feverish. She was evacuated to the clinic at RRC and presented with a high fever (40 °C), lethargy, dehydration, and findings of normocytic normochromic anaemia, leukopenia, thrombocytopenia and *P. pitheci* parasitaemia at 20,740/µL. She was diagnosed with acute malaria and administered ACT and other supportive treatments. Although she at first improved, five days after the start of the treatment her condition worsened with fever, lethargy and anorexia. Her haematology values remained low. An in-house blood culture revealed infection with *Pseudomonas spp.* She was administered a course of antibiotics and she fully recovered.

Case 10 (Melky): A sub-adult male orang-utan (approx. 12 years old) released into the wild 3 weeks earlier was found inactive, anorexic and febrile (39 °C). He was administered analgesics (paracetamol) and transported to the RRC for further medical attention. On arrival at the clinic, he was lethargic and vomiting with a productive cough. He was administered IV fluids and other supportive medication. No specific diagnosis was made but he remained under observation and supportive IV fluid-therapy. Four days after the onset of symptoms, blood film examination revealed *P. pitheci* parasitaemia at approximately 12,000/µL of blood. Blood biochemistry analysis also revealed hyperkalaemia and elevated creatinine levels. A possible renal disorder was suspected and since there was no clear sign of clinical malaria (i.e., no fever, anaemia, thrombocytopenia or leukopenia) the primary cause of his illness was unclear. He remained under observation and supportive therapy. The following day, his condition had not improved and new symptoms were detected such as fever (39.5 °C) and thrombocytopenia. The parasite load in blood had increased to 42,600/µL. Based on the new symptoms (fever and thrombocytopenia) and the increasing levels of *Plasmodium* in his blood, anti-malarial treatment (ACT) was initiated. Symptoms persisted for 48 h, with haemoglobin values dropping during this time. On day three after the initiation of the treatment, however, the fever remitted and he appeared fully recovered. By the end of the ACT treatment course, he had no parasitaemia and a normal blood profile.

Case 11 (Ceria): a sub-adult female orang-utan (approx. 12 years old) had experienced a minor surgery and was in the process of recovery. In the days post-surgery, she was lethargic and peaked a fever of 39.4 °C. Examination of her blood showed thrombocytopenia and a high parasitaemia load (over 25,000 pars/µL). She remained in observation. On the following day, her condition deteriorated, becoming more lethargic. Her fever peaked again (38.7 °C) and her blood sample revealed hyperparasitaemia (over 105,000 pars/µL). She was then administered supportive IV therapy (fluids and analgesics) and she was started on ACT. Fever remitted within 24 h post treatment and full recovery was reported in 72 h.

Case 12 (Maili): A sub-adult (approximately 12 years old) female orang-utan and mother to a 1-year-old infant, was being monitored in the wild after having been released 5 weeks prior to the onset of symptoms. She was found inactive and lethargic and was evacuated to the clinic at RRC. At physical examination, the patient presented a fever (38.6 °C) and slight dehydration. Further tests revealed normocytic normochromic anaemia, leukopenia and thrombocytopenia, and microscopic blood examination revealed a *P. pitheci* load of 3400/µL. Differential diagnoses included typhoid fever, malaria, and dengue fever, but all diagnostic tests conducted were inconclusive with regard to the cause of illness, so no final diagnosis was made. She was administered IV fluids and other supportive therapy, including analgesics/antipyretics, and remained in observation for 48 h. A new blood sample revealed that the parasite load had increased to 4680 pars/µL of blood. The increase in parasites and in severity of symptoms indicated malaria as the most probable cause of illness. ACT was administered together with other supportive therapy. After completing the course of antimalarial treatment, her condition and her appetite had improved and blood haematology values had increased, but she remained lethargic. At this stage, a positive *Salmonella* spp. test indicated a possible typhoid fever infection and she was placed on a course of antibiotics. At the end of the treatment, her condition as well as her haematology values were back to normal.

Case 13 (Susi): A sub-adult female orang-utan (approximately 13 years old), released into the wild 3 weeks before the onset of symptoms, was found lethargic, feverish and anorexic. After evacuation to the RRC, she was found to present a fever (38.8 °C), severe normocytic normochromic anaemia, leukopenia and thrombocytopenia, and a blood sample examination revealed a high level of *P. pitheci* in blood. ACT was administered together with antipyretics. Symptoms improved immediately after initial dose and a full recovery, including haematology values, occurred by the end of the treatment course (7 days).

Case 14 (Cinta): An adult female orang-utan over 15 years was reported lethargic, anorexic and feverish. Blood analysis revealed severe thrombocytopenia and *P. pitheci* parasites in her blood (25,000/µL). The following day the patient remained anorexic, lethargic and severely dehydrated. Further examination under sedation revealed a high fever (40.3 °C), a deepening thrombocytopenia, leukopenia, and a parasite concentration in her blood of 23,000/µL. She was started on ACT and administered supportive therapy. Fever remitted within the first 48 h after treatment. Four days after the initial antimalarial treatment, she presented active and with a normal appetite, and fully recovered before the end of the antimalarial treatment.

Case 15 (Nicky): An adult female orang-utan of approximately 15–17 years of age was found lethargic, febrile and anorexic. She could not be handled without sedation, so she was administered analgesics (paracetamol) and monitored for the following 48 h. She did not improve and so was sedated for a closer physical examination. She was shivering but did not present a fever. Microscopic examination of a blood sample revealed *P. pitheci* parasites at 9300/µL and blood analysis revealed severe anaemia, leukopenia and thrombocytopenia. She was administered supportive therapy and started on ACT. Her condition started improving within 24 h. She was considered fully recovered within a week.

Case 16 (Neng): An adult female orang-utan of about 17 years of age and suspected to be pregnant was found inactive, lethargic, anorexic and feverish. She was handled under sedation for further examination and her blood showed the presence of *P. pitheci* at a density of 14,000/µL. Haematology values also showed normocytic normochromic anaemia, thrombocytopenia and leukopenia. She was started on anti-malarial medication (ACT) and was administered IV fluids and other supportive medication. Potential early signs of pregnancy were identified. 48 h after the start of the treatment she still appeared lethargic and anorexic. She was sedated and administered supportive therapy again and started on a course of antibiotics. Two weeks after the first onset of the illness, her condition worsened. She was moved to intensive care and administered antibiotics and other IV therapies. 48 h later, her condition started to improve. After the course of antibiotics, she showed obvious improvement. Since no further signs of pregnancy were detected, a miscarriage possibly related to acute malaria was considered. She fully recovered within a month of the onset of symptoms.

#### Severe malaria

**Fig. 4:**
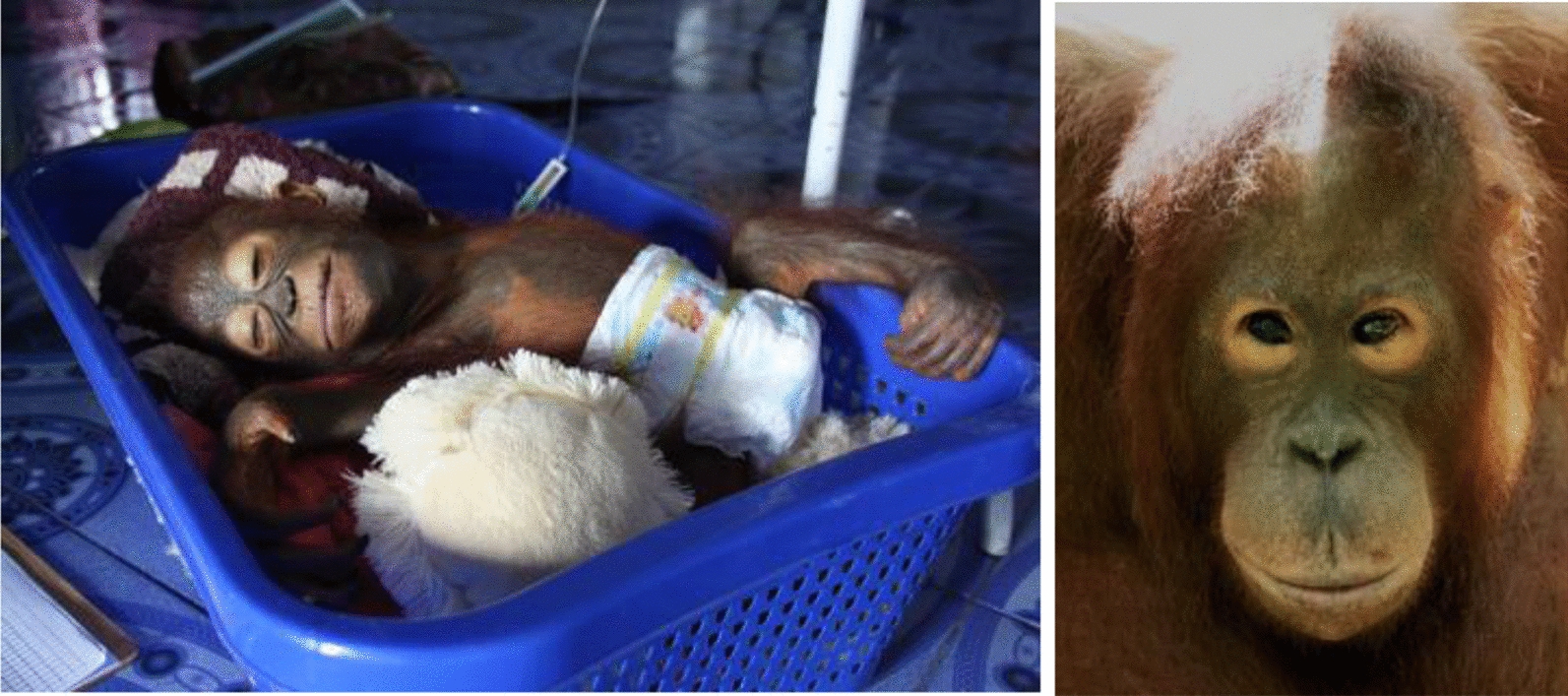
At left is Rahayu upon arrival at the RRC after rescue in 2011. The infant orang-utan presented with signs of cerebral malaria but recovered with anti-malarial therapy. At right in 2021, she is seen to have permanent strabismus in left eye resulting from her early malaria episode

Case 17 (Rahayu): An infant female orang-utan (age between 2 and 3 years old) was rescued by the authorities from a forest clearance area. She presented to the RRC febrile and prostrated with a low level of consciousness (Fig. 2). Examination of her blood on microscopy confirmed a patent *Plasmodium* spp*.* infection. The course of illness included fever (38.7 °C), multiple convulsions, nystagmus and other neurological impairments such as blindness and hearing loss, along with a mild anaemia. Treatment included intravenous (IV) fluids, anti-malarial treatment, analgesics, antibiotics, anti-convulsive and other supportive therapies. The first anti-malarial treatment consisted of oral quinine and doxycycline combination, but did not result in improvement within 48 h, so anti-malarial medication was switched to ACT. The most severe neurological signs remitted within 48-72 h after the start of the ACT, and hearing improved in the weeks after the severe clinical episode. Vision impairment did not improve and cortical blindness and strabismus remained as permanent sequelae of the infection (Fig. 4). In the years following rescue and the malaria episode, ophthalmological examination revealed macular whitening of the retina.

### Laboratory findings in clinical malaria cases

#### Haematology values:

Anaemia was diagnosed in 15 of 17 cases; leukopenia in 7 of 17 cases, and thrombocytopenia was reported in 12 of 17 cases. In all 17 cases at least one of these symptoms was recorded. Table 5 lists haematology values with clinical malaria. All are either below or at the lower range of normal.

**Table 5:** Blood haematology values in 17 malaria clinical cases using the lowest values during the convalescence period and compared to healthy range values obtained from the IAR population in Table 4

Analyte	Units	Range values	Mean	SD	Median	Range of normal values in orang-utans at IAR
Red blood cell count (RBC) (automated)	10^12^/L	1.20–4.80	3.13	0.95	3.22	3.11–6.29
Haemoglobin (HGB) (automated)	g/dL	2.9–10.10	6.63	1.86	7.00	8.2–13.4
Haematocrit (HTO) (automated)	%	9.26–32.06	20.37	7.72	21.60	23.8–43
Platelet count (PLT) (automated)	10^9^/L	2–237	62	68	37	70–385
White blood cell count (WBC) (automated)	10^9^/L	1.94–17.79	6.09	3.65	5.27	4.7–18.4

#### Parasitaemia and illness

**Fig. 5:**
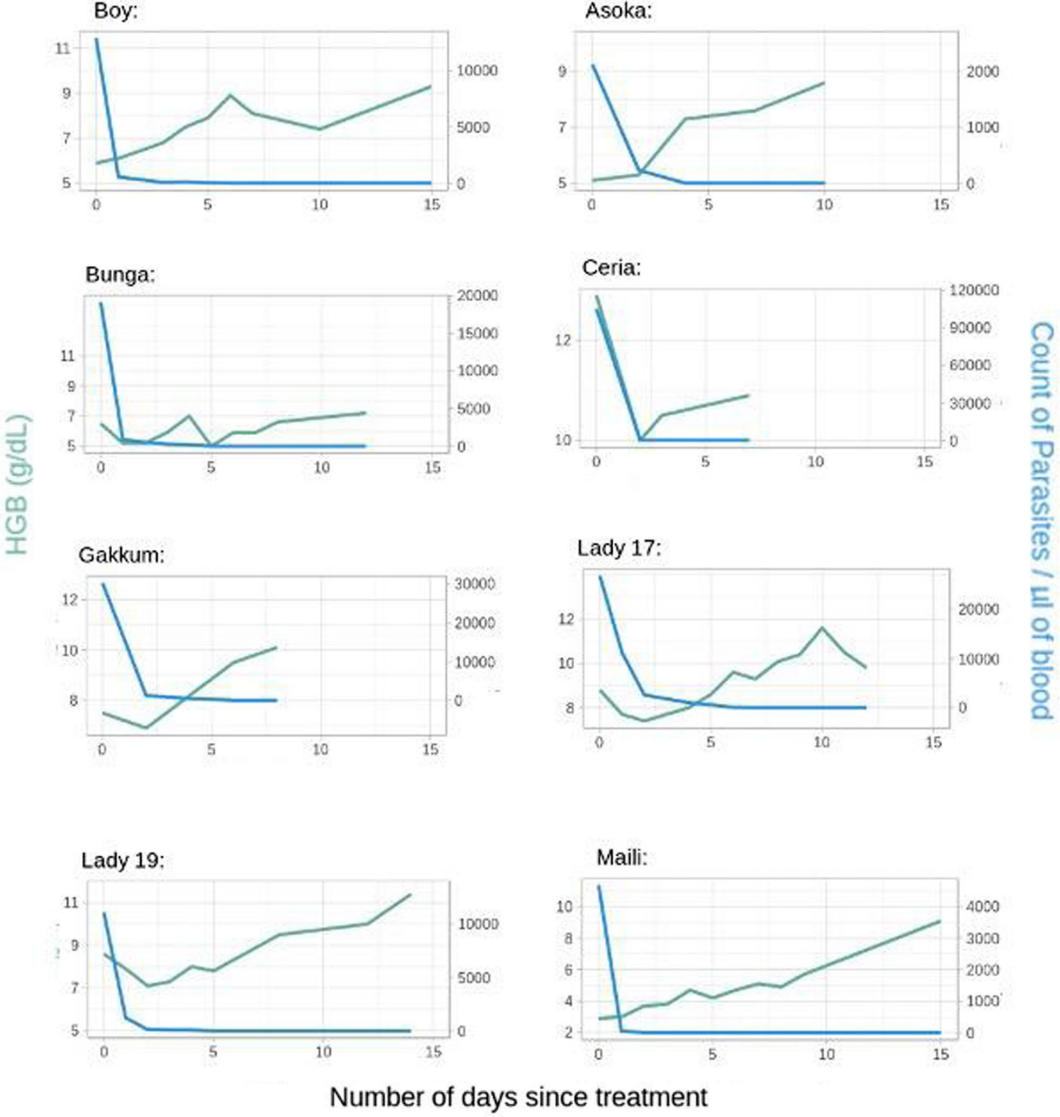
Graphics representing the trend of HGB values and parasitaemia in 8 acute and convalescent malaria cases receiving anti-malarial therapy

Statistical analysis was conducted to determine whether a correlation between parasite load and blood values of HGB (Fig. 5), PLT and WBC exists. The results showed that parasite load was negatively correlated with PLT (p < 0.001) values but not significantly correlated with WBC (p < 0.1). It was also noted that for HGB values, a positive correlation was highly significant (p < 0.001) at high parasite thresholds (> 2000 parasites/µL) but not significant at low parasite thresholds (p < 0.1), suggesting a regime in which parasitaemia levels correlate with a drop of HGB at high parasite loads but with the relationship disappearing once the number of parasite in blood lowers as effect of the anti-malarial medication (as it can be seen in Fig. 5). This relationship can be seen in the individual plots, in which HGB sometimes decreases with the initial parasite load decrease, before increasing (as in the case of Bunga, Ceria, Gakkum, and Lady’s two cases) (Fig. 5).

The detection limit of parasites in blood using microscopy is considered to be between 4 and 100 par/µL [15]. In our study, the lowest parasitaemia detected was 29 par/µL. Table 6 summarizes parasitological findings from symptomatic and asymptomatic infections by *P. pitheci*. Parasitaemia was measured in 14 clinical malaria cases and ranged between 4680 and 105,233/µL with a mean parasite density of 24,601 parasites/µL. In contrast, in 442 blood samples from 82 individuals obtained from asymptomatic malaria, the mean parasitaemia level was 956 parasites/µL with a maximum of 20,880/µL.

**Table 6:** Number of parasites in one microliter of blood in orang-utans with symptomatic and asymptomatic malaria

	Parasites/µL blood (asymptomatic)	Parasites/µL blood (symptomatic)
N	442	14
Mean	956	24,601
Max	20,880	105,233
Min	29	4680
Median	428	17,530
S.D	1840	24,948

### Treatment outcomes in clinical malaria cases 

**Fig. 6:**
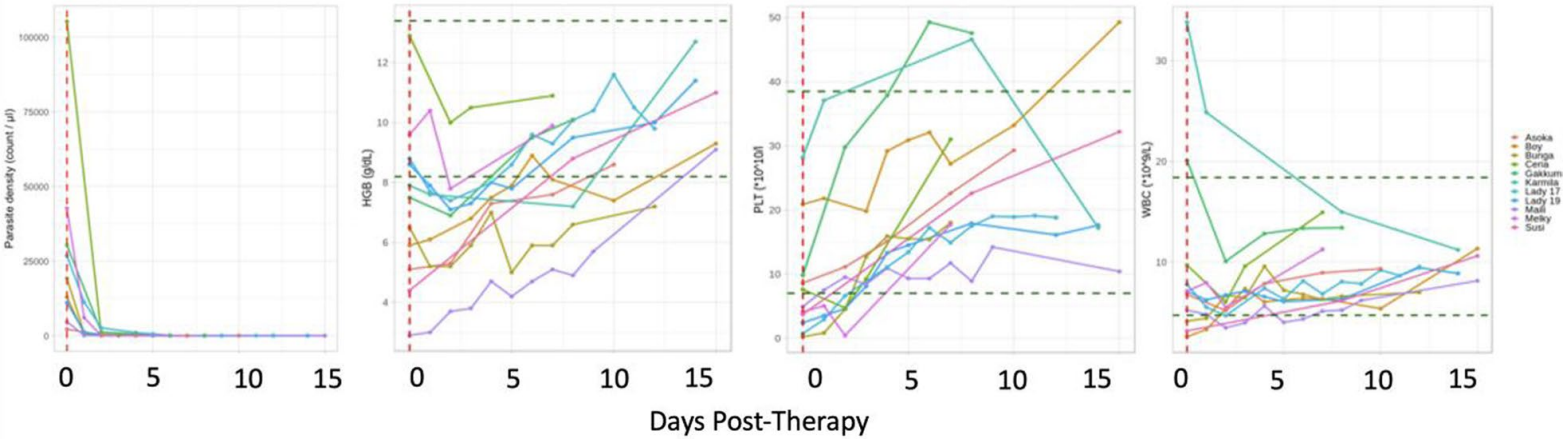
Trend of parasitaemia, HGB, PLT, and WBC values with clinical and convalescent malaria in orang-utans receiving anti-malarial treatment

15 clinical malaria patients received anti-malarial treatment while two others presented mild signs of malaria and experienced spontaneous reduction of parasites in blood and self-recovery without the need for anti-malarial treatment. All treated patients received ACT, although two of those first received quinine-doxycycline with an inadequate clinical response. The convalescence period ranged between 2 days (for both self-limiting cases) and 19 days with an average of 9 days (supplementary material). The maximum period required for HGB values to return to normal values ranged between 5 and 16 days (Figs. 5 and 6).

To evaluate the treatment response, values of each blood variable at the start and end of the treatment period were examined (7 days after the onset of ACT or at any other closer date). In the case of PLT, the mean value of PLT went up 7 days later as compared to the value at the start of treatment by a mean difference of 17.6 (*10^10/L) (p < 0.001). In the case of parasites in blood, the mean density of parasites went down 7 days later as compared to the value at the start of treatment by a mean difference of 27,795 parasite/µL of blood (p < 0.05). In the case of HCT (4.8% p < 0.05) and HGB (1.48 g/dL p < 0.05), the values went up 7 days later (Fig. 6).

As shown in Figs. 5 and 6, the trend in HGB, PLT and WBC values increases while the parasitaemia levels decrease from the onset of ACT and for up to 15 days post-treatment (until full recovery). In most cases, HGB values continued dropping after the start of anti-malarial treatment for up to 2.5–3 days to then increase. This might explain the highly significant positive correlation between parasite loads and HGB as explained above. Total clearance of parasites in blood was reported in all cases within 3 days of treatment.

## Discussion

The *Plasmodium* species routinely infecting orang-utans at IAR RRC was identified as *P. pitheci*. The morphological features of the parasites were consistent in all 466 positive blood films from 89 orang-utans in lacking enlargement of RBC and clumping of pigment, and in the absence of mature schizonts having > 14 merozoites. No evidence of the involvement of any other plasmodial species was found despite multispecies infections being commonly reported in African great apes [11, 12, 24, 32, 37, 61]. Among over 50 full-time staff in close contact with the orang-utans at the IAR RRC, no known case of malaria has occurred since 2015, making both anthroponotic and zoonotic malaria at this site highly improbable. The plasmodial infections in the orang-utans were highly prevalent and stable, appearing naturally adapted to this host and not being transmitted to humans despite prolonged close exposure to that apparently high risk. Indeed, *P. pitheci* is considered a host-specific parasite [60] and experimental infection with this parasite in humans and other primate were unsuccessful [23, 33]. The other known plasmodial species of orang-utans, *P. silvaticum*, would have been readily distinguished from *P. pitheci* by enlarged infected RBC, but this was never observed in any blood sample. Others have also reported *P. pitheci* in orang-utans without *P. silvaticum* being present [29, 34]. Despite the inability to execute definitive diagnosis by molecular methods, all the direct and indirect evidence gathered aligns with the diagnosis of *P. pitheci*, although the occurrence of an as-yet undescribed plasmodial species of orang-utans very closely resembling *P. pitheci* cannot be ruled out.

Stable endemic transmission of *P. pitheci* occurred at the IAR RRC over 5 years (2017–2021) since prospective active case detection started, and very probably since the beginning of operations over a decade earlier. The majority of instances of confirmed parasitaemia were not associated with any signs or symptoms of illness (452 of 466; 97%), apart from occasional mild anaemia. Nonetheless, moderate to severe and potentially life-threatening illness attributable to *P. pitheci* infection occurred among 13 of the 89 (14%) orang-utans known to have been infected. Three more clinical cases were detected between 2010 and 2016. These attacks involved febrile syndromes with anaemia, thrombocytopenia, leukopenia, anorexia, and lethargy. In one instance, cerebral malaria with permanent sequelae occurred in an infant. Although in the cerebral malaria case morphological identification of the species was not conclusive, it was not diagnosed as a human malaria parasite. A validated means of diagnosis and treatment of this apparently common illness in this endangered species would serve the cause of its conservation. This study strived to contribute to that goal.

Based on the experience reported here, orang-utan clinical pitheci malaria diagnostic criteria were developed. Clinical malaria may be affirmed by finding an increasing density of parasitaemia with an axillary temperature > 38 °C, moderate to severe normocytic normochromic anaemia (HGB below 8.2 mg/dL), and/or thrombocytopenia (PLT lower than 70*10^9^/L) and/or leukopenia (WBC below 4.7*10^9^/L), accompanied by other general symptoms including lethargy and/or anorexia. Potentially more severe syndromes involving vital organs such as kidney or brain may also occur. Some studies on human malaria estimated blood parasite density thresholds to define clinical malaria [15, 51, 62]. The mean parasite count from orang-utans with asymptomatic malaria was 956 par/µL, versus a mean of 24,601 par/µL in clinically ill patients (Table 6). The density threshold of parasitaemia in symptomatic cases was estimated at 4000/µL. The clinical experience obtained at this RRC confirms that acute malaria in orang-utans occurs and is quickly resolved with ACT administration.

A case of severe malaria in an infant orang-utan which resembled cerebral malaria in humans was described (case 17). There was fever, anaemia and signs of neurological impairment (prostration, nystagmus, impaired hearing, blindness and multiple convulsions), along with cortical blindness and strabismus remaining as sequelae of the illness. The macular whitening of the retina which was diagnosed in this patient a decade after the acute illness also occurs in human patients surviving cerebral malaria [62–65] and is considered a diagnostic sign for this illness [66]. These findings support the evidence for a severe case of cerebral malaria in this infant orang-utan. To our knowledge, this is the first confirmed case of cerebral malaria in an orang-utan or any other great ape, and it highlights the potential severity and even fatality caused by this illness if going untreated.

One adult female orang-utan (case 16) may have suffered a miscarriage during her acute malaria attack. In humans, placental malaria can lead to miscarriage, low birth weight, peri-partum haemorrhage or increased maternal and neonatal mortality, amongst others [1, 5, 58]. While we cannot confirm that this pregnancy failed as a direct result of malaria, it is nonetheless plausible. De Nys et al. reported higher malaria parasite detection rates during pregnancy in a population of chimpanzees [26]. Any threat to the reproductive health of orang-utans (or other great apes) as a consequence of a highly prevalent endemic infectious disease merits close attention and further examination.

It may be overly optimistic to dismiss the health impact of the majority of *P. pitheci* infections as inconsequential, especially when wild orang-utans, alike the captive population, would go untreated for malarial symptoms. In one of the patients (case 3), a chronic microcytic and hypochromic regenerative anaemia which had not responded to iron supplementation, had been diagnosed prior this malaria episode. This patient had also been found to present high morbidity due to different illnesses and had poor general health before the confirmed malaria episode. Following anti-malarial treatment his anaemic condition resolved and no further episodes of sickness were reported post treatment and for the following five years. The relationship between such poor general health and chronic malaria in otherwise asymptomatic plasmodial infections may be confounded by many factors [56, 58, 67]. However, it is believed that chronic patent malaria infections can be accompanied by other sub-clinical effects in humans [15, 58] and several studies conducted in children have looked at potentially detrimental health effects of what are widely considered asymptomatic infections [58]. If chronic malaria infections negatively impact the general health of orang-utans, then diagnosis and treatment of any malaria in the RRCs may need to be considered part of a conservation strategy for the optimal health of these populations.

Many of the orang-utans were followed for up to 5 years and were repeatedly positive for *P. pitheci* (Fig. 3). Whether these represented stable chronic infection or repeated spontaneous clearance followed by reinfection or relapse (hypnozoites of *P. pitheci* are not known) cannot be determined from this data. However, a patent *P. pitheci* infection was observed in an orang-utan upon rescue from a captive situation on another island who had been living in a malaria-free urban area without contact with other orang-utans for over 10 years. Peters et al. reported ‘persistent’ infections with *P. pitheci* in orang-utans lasting at least seven years [33]. Very long-term parasitaemia without spontaneous clearance is thus at least plausible.

The study findings confirm deleterious effects of natural malaria in orang-utans. In chimpanzees, two clinical cases of malaria caused by *Plasmodium reichenowi*, a species known to naturally infect these apes, have been reported [30, 39, 40]. While the pathogenicity of species naturally infecting chimpanzees continues to be debated [24], these findings support the notion that *Plasmodium* spp*.* infection of great apes could indeed be harmful in their natural host [24]. The most severe case documented in this study was recorded in an infant orang-utan. Similarly, mortality and other severe forms of malaria in humans occur mostly in children, as well as in pregnant women [1, 2, 4, 60]. However, while malaria causes mortality in the hundreds of thousands annually [1], only a very small proportion of the infected (hundreds of millions) progress to severe illness in endemic areas [4]. Most people are indeed protected from severe outcomes of infection through clinical interventions, but also by naturally acquired immunity to malaria. The same is likely true of *P. pitheci* in orang-utans, i.e., a non-sterilizing immunity that sometimes fails to curb progression of the infection and illness, especially in the very young. Since malaria cases diagnosed at RRCs are likely to be clinically managed, estimating potential mortality rates of this illness is not possible. However, infant mortality rates in wild orang-utan populations are reportedly higher than those of other great ape species [28]. Pitheci malaria may be involved in that mortality.

## Conclusions

This study spans a decade of observations in caring for orang-utans in rescue and rehabilitation at a forested IAR RRC preserve in West Kalimantan, Indonesia. The study findings confirm the stable endemic presence of *P. pitheci* on that preserve and the onset of moderate to severe illness in 14% of infected orang-utans. Acute malaria typically presented with fever, anaemia, thrombocytopenia, leukopenia, and lethargy with sometimes anorexia in association with relatively high parasitaemia. A case of cerebral malaria with permanent neurological sequelae is also reported. The successful diagnosis, clinical management, and anti-malarial chemotherapy of acute pitheci malaria in orang-utans are described. Contrary to the conventional view of natural *P. pitheci* infection as benign and inconsequential to the health of *Pongo pygmaeus*, this study confirms this parasite as the cause of sometimes serious illness at the IAR RRC. This endemic infection did not infect humans despite prolonged high risk of exposure among IAR RRC staff. As no human malaria occurred at this site, it is not confirmed if anthroponotic infections of orang-utan may occur. In summary, *P. pitheci* poses an endemic threat to the health of orang-utans in rescue and rehabilitation settings, as may also occur in the wild.

## Data Availability

The datasets generated and/or analysed during the current study are not publicly available due to being owned by the Indonesian Government but are available from the corresponding author on reasonable request.
